# Genome-Wide Identification of the ABF/AREB/ABI5 Gene Family in *Ziziphus jujuba* cv. Dongzao and Analysis of Its Response to Drought Stress

**DOI:** 10.3390/genes16070785

**Published:** 2025-06-30

**Authors:** Zhikai Zhang, Xiaoming Liu, Yu Wang, Jun Zhou, Zhongwu Wan, Xin Zhang, Jing Wang, Binbin Si, Lan Luo, Wendi Xu

**Affiliations:** 1 School of Biological Science and Engineering, North Minzu University, Yinchuan 750021, China; zhangzhikai0019@163.com (Z.Z.); 19908106585@163.com (X.L.); zhoujunbo@163.com (J.Z.); x_zhang@nmu.edu.cn (X.Z.); wangjing_imu2@163.com (J.W.); sibinbin115@163.com (B.S.); luolan020313@163.com (L.L.); 2 Ningxia Lingwu Horticultural Farm, Lingwu 750400, China; nihao_826@163.com; 3 Innovation Team for Genetic Improvement of Economic Forest, North Minzu University, Yinchuan 750021, China; 4 Daquan Forestry Centre of Lingwu City, Lingwu 750400, China; nxlwbsw@163.com; 5 Lingwu Baijitan Natural Resources Conservation Bureau, Lingwu 750400, China

**Keywords:** *ABF/AREB/ABI5* gene, genome-wide analysis, abscisic acid, drought stress, gene expression, *Ziziphus jujuba* cv. Dongzao

## Abstract

Abscisic acid (ABA), a pivotal phytohormone regulating plant growth and stress adaptation, orchestrates abiotic stress responses through the ABA-responsive element-binding factors ABF/AREB/ABI5. Nevertheless, the functional characterization of ABF/AREB/ABI5 homologs in *Z. jujuba* cv. Dongzao remains unexplored. In this study, we identified seven *ZjABF* genes distributed across five chromosomes. Domain analyses revealed high structural conservation, particularly within the basic leucine zipper (bZIP) DNA-binding domain. Subcellular localization confirmed nuclear targeting of all seven ZjABF proteins. Phylogenetic classification resolved these factors into three clades (A–C). Cis-regulatory element profiling implicated the involvement of the ZjABFs in hormone signaling, abiotic stress transduction, and photoregulatory pathways. Synteny analyses identified three segmental duplication events within the gene family. Tissue-specific expression patterns indicated critical roles for *ZjABF2* and *ZjABF3* in fruit maturation, and most of the *ABF/AREB/ABI5* genes were highly expressed in the root. Under drought stress, four *ZjABF* genes exhibited differential expression, with *ZjABF2* demonstrating pronounced sensitivity. These findings establish a molecular framework for understanding *ZjABF*-mediated abiotic stress responses in non-model woody perennials.

## 1. Introduction

*Z. jujuba* cv. Dongzao constitutes a vital cash crop across northwestern China’s semi-arid regions, being renowned for its exceptional nutritional profile and commercial significance [1]. Lingwu City in Ningxia—a primary production hub for *Z. jujuba* cv. Dongzao—faces chronically hyper-arid conditions characterized by deficient precipitation (<200 mm annual mean) and excessive evaporation (>2000 mm annual mean) [2]. These abiotic constraints severely limit both the yield and quality of fruit. Drought significantly reduces fruit yield and quality, decreasing individual fruit weight by 25–30%, lowering fruit set rates by 20–40%, and diminishing per-hectare production by over 25%, while also impairing peel coloration, reducing storability, and disrupting sugar–acid balance. Recent climate change has amplified the threats posed by drought stress to agricultural productivity globally, making the dissection of *Z. jujuba* cv. Dongzao’s drought resilience mechanisms imperative for safeguarding regional agricultural sustainability [3]. Analyzing the drought-resistant molecular mechanism of *Z. jujuba* cv. Dongzao has become the key to ensuring the sustainable development of regional agriculture.

Plants deploy intricate transcriptional networks to orchestrate their adaptation to various stresses [4]. Central to these networks, ABF (ABRE-binding factor) genes—core members of the bZIP transcription factor family—function as master regulators by binding ABA-responsive elements (ABREs) to activate downstream stress-responsive genes. Also designated AREBs (ABA-responsive element-binding proteins) or ABFs (ABRE-binding factors), these proteins play non-redundant roles in abiotic stress resilience, notably in drought and salinity tolerance [5,6]. The ABF family exhibits broad phylogenetic conservation across plant lineages, though the number of members varies significantly (5–36 homologs) between species, such as Arabidopsis, rice (*Oryza sativa*) [7], wheat (*Triticum aestivum*) [8], and *Z. jujuba* [9]. Structurally, ABF proteins contain four conserved kinase-targeting domains (C1–C4): N-terminal C1–C3 motifs mediate signalosome interactions, while the C-terminal C4 domain stabilizes the tertiary structure through α-helix maintenance [10].

ABF family members exhibit pronounced specificity of spatiotemporal expression, enabling modular transcriptional programs during plant development. For instance, in tomato (*Solanum lycopersicum*), *SlABF2* and *SlABF10* display significant upregulation across fruit ripening stages [11]; apple (*Malus domestica*) orthologs predominantly accumulate in leaf tissues [12]; and wheat shows differential partitioning. *TaABF5A*, *TaABF5B*, and *TaABF5D* are ubiquitously expressed in seedling roots, stems, and leaves, whereas *TaABF5A/B/D* and *Ta ABF18-B/D* localize specifically to roots and leaves [8]. Collectively, these findings demonstrate ABF-mediated developmental regulation through tissue-specific transcriptional specialization.

*ABF* family genes function as multimodal stress sensors, dynamically activating ABA signaling cascades to coordinate downstream stress-responsive gene networks. This regulatory axis mediates adaptation to drought through three core physiological modules: modulation of stomatal closure, biosynthesis of osmoregulatory solutes, and reactive oxygen species (ROS) scavenging via antioxidant enzyme activation. Some studies have analyzed and demonstrated the important roles of the ABF/AREB/ABI5 family in enhancing the non-biological stress adaptability and resistance of rice [7], wheat [6], potato [6], sweet potato [13], cotton [14], and strawberry [15]. For example, in wheat, *TaABF17-19* is significantly upregulated under drought and salt stress [8]; ten *SlABF*s in tomato can respond to ABA induction; *SlABF3* and other genes are significantly upregulated under salt and cold stress [11]; in apple, *MdABF1* is significantly upregulated under drought stress, such that overexpression of *MdABF1* enhances drought resistance [12]; overexpression of Arabidopsis *AtABF3* activates *RD29B*, significantly enhancing plant drought resistance [16]; and, in rice, *OsABF1* enhances drought resistance by regulating the expression of *COR413-TM1* and other genes [17]. However, as a characteristic tree species in arid areas, the systematic identification of *ABF* genes, their evolutionary features, and drought response mechanisms in *Z. jujuba* cv. Dongzao has not yet been achieved, limiting the precision of its molecular breeding.

Consequently, our genome-wide characterization of the ABF transcription factor family in *Z. jujuba* cv. Dongzao, integrating phylogenetic profiling, structural bioinformatics, and spatiotemporal expression mapping, establishes a mechanistic framework for abiotic stress resilience in this economically vital crop. This work further delivers actionable genetic targets for molecular breeding programs aimed at enhancing drought tolerance.

## 2. Materials and Methods

### 2.1. Materials

The experimental materials for RNA extraction, cDNA synthesis, fluorescent quantitative analysis and drought stress treatment were cultivated at the Daquan Forestry Centre of Lingwu City (106°3′32″ E, 39°57′65″ N), at an altitude of 1250 m. The experimental trees were planted in plastic greenhouses with a row spacing of 3 m × 4 m, a temperature of 18–32 °C (night/day), and a photoperiod of 14 h. The soil was sandy loam, deep and fertile, and was irrigated using Yellow River water. The average annual temperature in the region is 9.6 °C, with an average annual sunshine duration of 3002.1 h, an average frost-free period of 157 days, and an annual accumulated temperature of 3351.3 °C. Annual precipitation ranges from 179.2 to 322.4 mm.

Plant samples from different parts (young leaves, old leaves, young stems, old stems, flowers, and roots) were collected in May, and fruit samples from different stages (white ripe stage, half-red stage, and fully red stage) were harvested from June to September. The samples were quickly placed in liquid nitrogen and stored at −80 °C in a freezer.

The experiment was conducted from July to August 2024. The selected tree materials were all healthy, well grown, disease-free, and uniformly vigorous 8-year-old *Z. jujuba* cv. Dongzao trees with *Z. jujuba* var. spinosa (Bunge) Hu ex H.F. Chow as the rootstock, and the row spacing measured 2 m × 4 m. Four treatments were set up: normal irrigation treatment (about 10 kg of water every three days, denoted CK), T1 treatment (light drought stress treatment, about 7.5 kg of water every three days, denoted LD), T2 treatment (moderate drought stress treatment, about 5 kg of water every three days, denoted MD), and T3 treatment (severe drought stress treatment, about 2.5 kg of water every three days, denoted HD). The soil’s relative moisture content was monitored and controlled using a hygrometer. We performed three biological replicates for each treatment, with five cycles over a total of 15 days. For sampling, randomly selected *Z. jujuba* cv. Dongzao fruits without mechanical damage and exhibiting consistent developmental stages were collected, wrapped in aluminum foil, and stored in liquid nitrogen before being transported back to the laboratory and preserved in an ultra-low-temperature freezer.

### 2.2. Research Methods

#### 2.2.1. Identification of the *Z. jujuba* cv. Dongzao ABF/AREB/ABI5 Gene Family

A total of 9 AtABF protein sequences were downloaded from the Arabidopsis database (https://www.arabidopsis.org/ (accessed on 17 April 2024)) (*AtABF1 (At1g49720*), *AtABF2/AREB1* (*At1g45249*), *AtABF3* (*At4g34000*), *AtABF4/AREB2* (*At3g19290*), *AtABI5/DPBF1* (*At2g36270*), *AtDPBF2* (*At3g44460*), *AtDPBF3/AREB3* (*At356850*), *AtDPBF4* (*At2g41070*) and *AtbZIP15* (*At5g42910*)) as search sequences. The genomic and proteomic data of *Z. jujuba* cv. Dongzao was downloaded from NCBI (https://www.ncbi.nlm.nih.gov/ (accessed on 17 April 2024)), followed by alignment, screening, and identification. Secondly, an E-value of 1e-20 was used to reduce the false positive, and the PFAM database (http://pfam.xfam.org/ (accessed on 17 April 2024)) and SMART database (http://smart.embl-heidelberg.de/ (accessed on 17 April 2024)) were used to further verify the ABF/AREB protein domain of *Z. jujuba* cv. Dongzao. Candidate genes that did not contain a specific domain of the *ABF/AREB/ABI5* gene (cd14707) were manually eliminated.

#### 2.2.2. Physicochemical Property Analysis of the *Z. jujuba* cv. Dongzao ABF/AREB/ABI5 Gene Family

The online website ExPASy was used (https://www.expasy.org/ (accessed on 18 April 2024)) to analyze and statistically predict the physicochemical properties of the identified *Z. jujuba* cv. Dongzao ABF/AREB/ABI5 family member proteins, including the number of amino acids, molecular weight, theoretical isoelectric point, and instability coefficient.

#### 2.2.3. *Z. jujuba* cv. Dongzao ABF/AREB/ABI5 Gene Family Subcellular Localization Prediction

The validated *Z. jujuba* cv. Dongzao ABF/AREB/ABI5 protein sequences were submitted to the online software Wolf PSORT (https://wolfpsort.hgc.jp/ (accessed on 18 April 2024)) for subcellular localization prediction [18].

#### 2.2.4. *Z. jujuba* cv. Dongzao ABF/AREB/ABI5 Gene Family Chromosome Localization

The gff gene annotation file of *Z. jujuba* cv. Dongzao was downloaded from NCBI to explore the chromosome location information of each gene. Using the *Z. jujuba* cv. Dongzao whole-genome sequence and gene structure annotation information, a chromosome localization map of the *Z. jujuba* cv. Dongzao *ABF/AREB/ABI5* gene family was drawn using TBtools 1.047 [19].

#### 2.2.5. Analysis of the Conserved Structural Domains and Motifs in the *Z. jujuba* cv. Dongzao ABF/AREB/ABI5 Family

MEME (http://meme-suite.org/tools/meme, accessed on 18 April 2024) was used to analyze conserved motifs in the *Z. jujuba* cv. Dongzao ABF/AREB/ABI5 transcription factor family by inputting all protein sequences into the online ABF/AREB/ABI5 analysis program [20]. The number of motifs was set to 7, while other parameters were set to default values. The *Z. jujuba* cv. Dongzao family was subjected to multiple sequence alignment using DNAMAN V6 software.

#### 2.2.6. Analysis of Gene Structure in the *Z. jujuba* cv. Dongzao ABF/AREB/ABI5 Family

Mega 11 was used to perform multiple sequence alignment on Arabidopsis and 7 of the *Z. jujuba* cv. Dongzao ABF/AREB/ABI5 protein sequences. A phylogenetic tree was constructed using the maximum likelihood method and was further modified using ITOL (https://itol.embl.de/itol.cgi, accessed 19 April 2024) for better visualization [21]. TBtools software was used to analyze the gene structure of the *Z. jujuba* cv. Dongzao *ABF/AREB/ABI5* gene family members.

#### 2.2.7. *Z. jujuba* cv. Dongzao ABF/AREB/ABI5 Protein Secondary and Tertiary Structure Prediction

SOPMA (http://npsa-pbil.ibcp.fr (accessed on 20 April 2024)) was used to predict the secondary structures of the *Z. jujuba* cv. Dongzao ABF/AREB/ABI5 protein sequences. In addition, the online website Swiss-Model (https://robetta.bakerlab.org/, accessed on 20 April 2024) was used to draw the three-dimensional structures of the different *Z. jujuba* cv. Dongzao ABF/AREB/ABI5 protein sequences.

#### 2.2.8. *Z. jujuba* cv. Dongzao ABF/AREB/ABI5 Gene Promoter Cis-Regulatory Element Analysis

Tbtools was used to extract the nucleotide data of the first 2000 bp of the *ABF/AREB/ABI5* gene family promoter region of *Z. jujuba* cv. Dongzao, and the cis-acting elements of the ABF/AREB/ABI5 promoter region of *Z. jujuba* cv. Dongzao were predicted using the online website PlantCARE (http://bioinformatics.psb.ugent.be/, accessed on 2 May 2024). After the results were obtained, they were input into Tbtools for visual analysis of the cis-acting elements [22].

#### 2.2.9. *Z. jujuba* cv. Dongzao ABF/AREB/ABI5 Gene Family Collinearity Analysis

Collinearity analysis was conducted using MCScanX, retaining syntenic blocks with at least 3 gene pairs. Duplication events were estimated using Ks values, with segmental duplication defined as Ks < 0.5 and syntenic block length ≥ 10 kb. The results were visualized using TBtools, with tandemly duplicated gene clusters highlighted in red.

#### 2.2.10. *Z. jujuba* cv. Dongzao ABF/AREB/ABI5 Protein Interaction Prediction

String (https://cn.string-db.org/, accessed on 5 May 2024) was used for protein interaction prediction analysis, with Arabidopsis thaliana set as the model plant.

#### 2.2.11. Synthesis of cDNA and qRT-PCR

Plant RNA Kit (Tiangen Biotech Co., Nanjing, China.) was used to obtain the RNA of *Z. jujuba* cv. Dongzao, which was extracted from the plants under abiotic stress and the young leaves, old leaves, young stems, old stems, flowers, roots, white mature fruits, half-red fruits, and all-red fruits of untreated plants. According to the instructions of the HiScript II 1st Strand cDNA Synthesis Kit (Vazyme, Nanjing, China), RNA was reverse-transcribed into cDNA.

Additionally, qRT-PCR was performed on a LightCycler 480 II Real-Time PCR System (Roche, Indianapolis, IN, USA), which is based on the SYBR Green (Sangon, Shanghai, China) method. The Primer 3 online tool was used to design specific primers of *ZjABFs* [23]. All reactions were performed for three biological replicates and four technical repeats.

#### 2.2.12. Data Analysis

We used the 2^−∆∆Ct^ method to acquire the gene expression in Microsoft Excel 2020 and analyzed whether there were significant differences in gene expression via SPSS Statistics 27 [24]. The significance of *ZjABF* gene expressions was analyzed by a one-way ANOVA.

## 3. Results

### 3.1. Identification of the Z. jujuba cv. Dongzao ABF/AREB/ABI5 Gene Family Members

**Figure 1:**
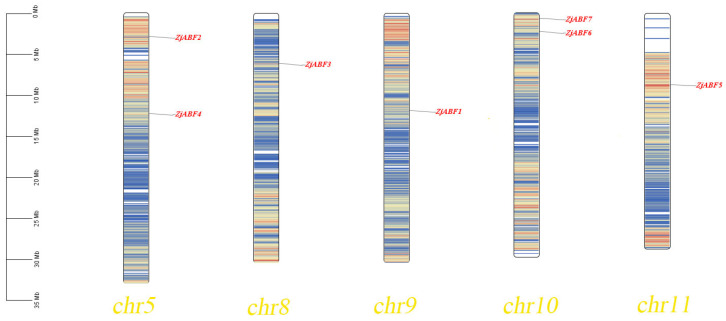
Chromosome location map of *ZjABF* family of *Z. jujuba cv. Dongzao.* The chromosome location map shows the location of seven *ZjABF* genes and the chromosome length unit is Mb.

Homology analysis identified seven *ABF/AREB/ABI5* genes in *Z. jujuba* cv. Dongzao. The presence of the conserved bZIP_plant_BZIP46 domain (cd14707) within these genes was confirmed using the Pfam, SMART, and NCBI Conserved Domain Databases. The genes were designated *ZjABF1* to *ZjABF7*. Chromosomal localization analysis, performed using TBtools software (v2.016), revealed an uneven distribution of the *ABF/AREB/ABI5* gene family members across five of the *Z. jujuba* cv. Dongzao chromosomes (Figure 1): *ZjABF2* and *ZjABF4* reside on Chr-5, *ZjABF3* on Chr-8, *ZjABF1* on Chr-9, *ZjABF6* and *ZjABF7* on Chr-10, and *ZjABF5* on Chr-11.

Analysis of the physicochemical properties of the ZjABF proteins revealed amino acid lengths ranging from 288 residues (ZjABF6) to 474 residues (ZjABF7) (Table 1), with corresponding molecular weights spanning from 32.46 kDa (ZjABF6) to 52.64 kDa (ZjABF7). The theoretical isoelectric points (pI) varied substantially from 5.39 (ZjABF4) to 9.85 (ZjABF1). Notably, only ZjABF4 was predicted to be an acidic protein (pI < 7), whereas the others exhibited basic properties (pI > 7). Stability assessment classified ZjABF2 as the sole stable protein within the family; the instability indices of the remaining six ZjABF proteins exceeded 40, categorizing them as unstable. The grand average of hydropathicity (GRAVY) values, reflecting lipid solubility propensity, ranged between 63.88 (ZjABF5) and 80.12 (ZjABF4). All ZjABF proteins exhibited negative GRAVY values, indicating their overall hydrophilic character. Subcellular localization predictions using WOLF PSORT consistently indicated nuclear localization for all seven ZjABF proteins (ZjABF1–ZjABF7), suggesting their involvement in processes related to genetic material storage and replication.

### 3.2. Conserved Domains and Conserved Motifs of the Z. jujuba cv. Dongzao ABF/AREB/ABI5 Family

**Figure 2:**
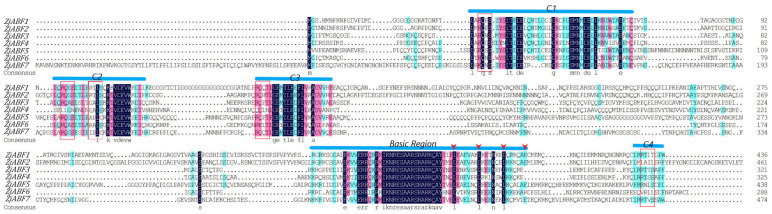
Multiple sequence alignment of Dongzao ABF/AREB/ABI5 members. The residues are shaded in pink, light blue, and dark blue. The positions from C1 to C4 are conserved domains, and the basic region is represented by the line above the protein sequence. The potential phosphorylation residues (R-S-SX/T) of characteristic phosphorylation sites are represented by red boxes. The conserved Leu residues are positioned in Leu zippers. Domains are represented by red triangles.

Multiple sequence alignment of the seven *Z. jujuba* cv. Dongzao *ABF/AREB/ABI5* genes, performed using DNAMAN V6, revealed conserved N-terminal regions containing three characteristic domains (C1, C2, C3) and C-terminal regions comprising a C4 domain coupled with a canonical basic region/leucine zipper (bZIP) module (Figure 2). The ABF/AREB/ABI5 domains harbor highly conserved sequences, including potential phosphorylation sites conforming to the R-X-X-S/T motif. The C-terminal BRLZ domain (core bZIP region) is implicated in the specific recognition of the ABA-responsive element (ABRE) cis-acting element.

**Figure 3:**
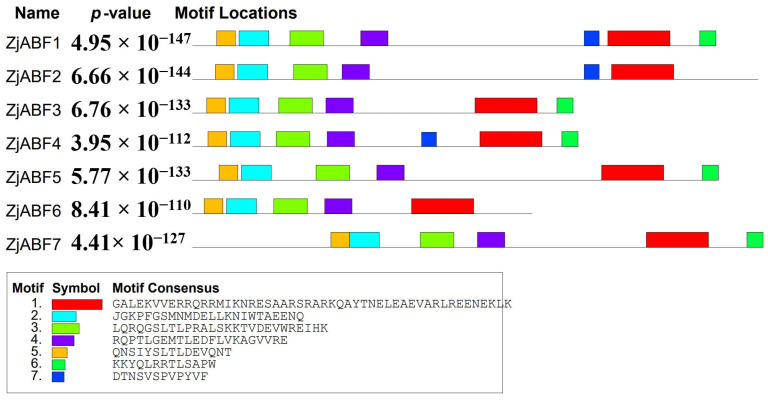
Sequence analysis of *ABF/AREB/ABI5* gene family in *Z. jujuba cv. Dongzao*. Differently colored rectangles represent different motifs.

To further characterize the gene family members, conserved motifs within the seven ZjABF proteins (ZjABF1–ZjABF7) were predicted using the MEME online tool. Analysis identified seven conserved motifs ranging in length from 12 to 50 amino acids (Figure 3), exhibiting high similarity. Core regulatory elements—specifically the bZIP domain and phosphorylation sites—were highly conserved across all family members, suggesting potential functional redundancy. Motif 1 constitutes the fundamental unit of the bZIP domain. The phosphorylation regulatory network appears complete, formed by Motif 5 and Motif 2 (corresponding to the C1 site), Motif 3 (C2 site), Motif 4 (C3 site), and Motif 6 (C4 site). Notably, Motif 7 is exclusively present in *ZjABF1*, *ZjABF2*, and *ZjABF4*, potentially conferring distinct regulatory properties to these members.

### 3.3. Z. jujuba cv. Dongzao ABF/AREB/ABI5 Family Gene Structure Analysis

**Figure 4:**
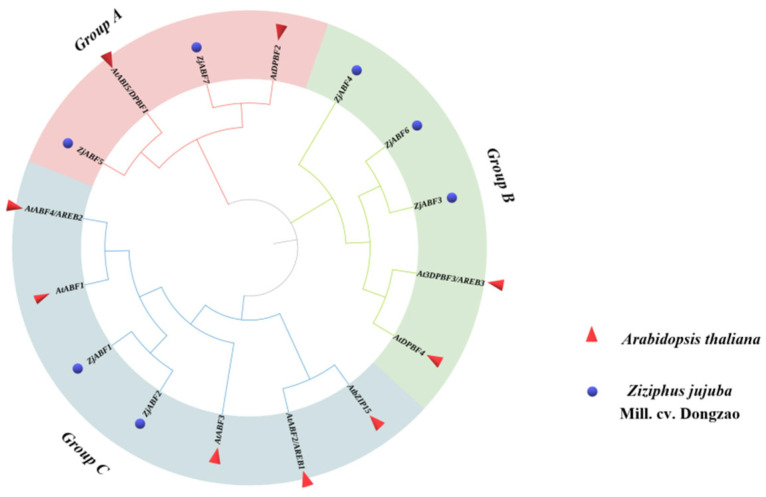
Phylogenetic relationships of AREB/ABF/ABI5 proteins in *A. thaliana* and *Z. jujuba* cv. Dongzao that were established by the neighbor-joining (NJ) connection method in MEGA X. The red triangle indicates *AtABF*s, and the blue circle indicates *ZjABF*s.

Phylogenetic analysis of the *Z. jujuba* cv. Dongzao ABF/AREB/ABI5 family, based on comparison with the amino acid sequences of nine *Arabidopsis thaliana* ABF/AREB/ABI5 proteins, revealed the evolutionary relationships within this group (Figure 4). The analysis resolved the 16 ABF/AREB/ABI5 proteins (7 *ZjABF*s from *Z. jujuba* cv. Dongzao and 9 *AtABF*s from A. thaliana) into three distinct subfamilies (designated Group A, Group B, and Group C). *ZjABF5* and *ZjABF7* clustered within Group A; *ZjABF4*, *ZjABF6*, and *ZjABF3* belonged to Group B; and *ZjABF1* and *ZjABF2* were assigned to Group C. Notably, *ZjABF7* and *ZjABF5* formed a clade with the Arabidopsis orthologs *AtDPBF2* and *AtABI5/DPBF1*, suggesting potential functional conservation between these genes.

**Figure 5:**
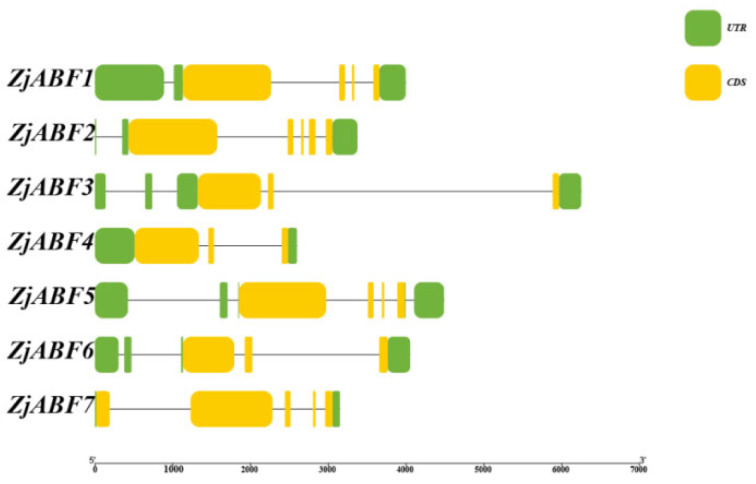
Schematic diagram of *ZjABF* family structure. These include CDS, UTRs, and introns.

Analysis of gene structure demonstrated that members of the *Z. jujuba* cv. Dongzao *ABF/AREB/ABI5* gene family contain between three and five exons and two to six introns (Figure 5). While the non-coding regions and the position and length of introns exhibited considerable variation among different family members, the exon structure and length within the coding regions were conserved.

### 3.4. Z. jujuba cv. Dongzao ABF/AREB/ABI5 Tertiary and Secondary Structure Prediction

**Figure 6:**
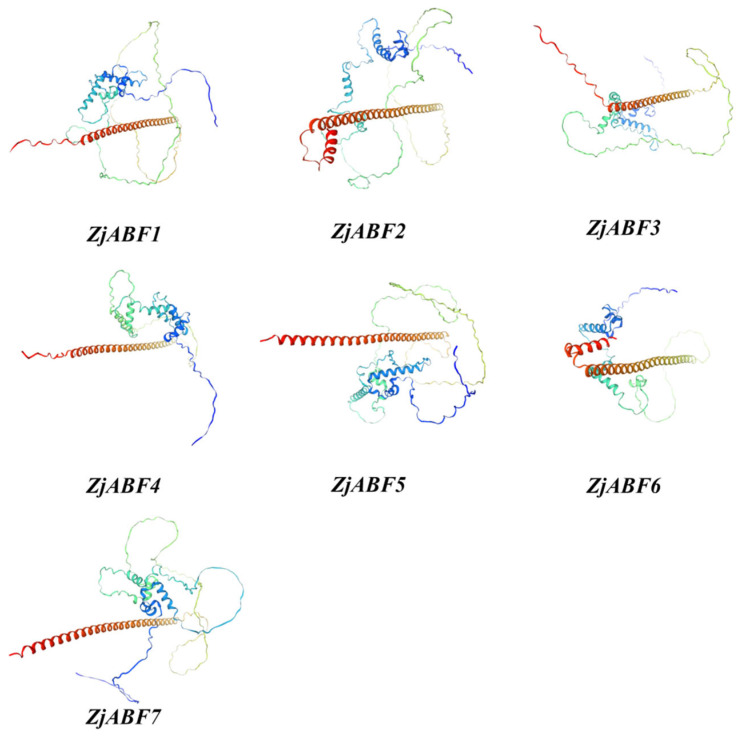
A schematic diagram of the tertiary structures of the ZjABF family of proteins.

Secondary structure prediction for the *Z. jujuba* cv. Dongzao ABF/AREB/ABI5 protein family members, performed using SOPMA, revealed that α-helices and random coils constitute the predominant structural elements (Table 2). Specifically, α-helices account for 28.21% to 46.88% of the residues, while random coils represent 50.35% to 71.10%. Extended strands constitute a minor component, ranging from 0% to 4.43%. Tertiary structure modeling of the ZjABF proteins using Swiss-Model (Figure 6) further supports this secondary structure composition.

### 3.5. Analysis of Cis-Acting Elements in Promoters of Z. jujuba cv. Dongzao ABF/AREB/ABI5 Genes

**Figure 7:**
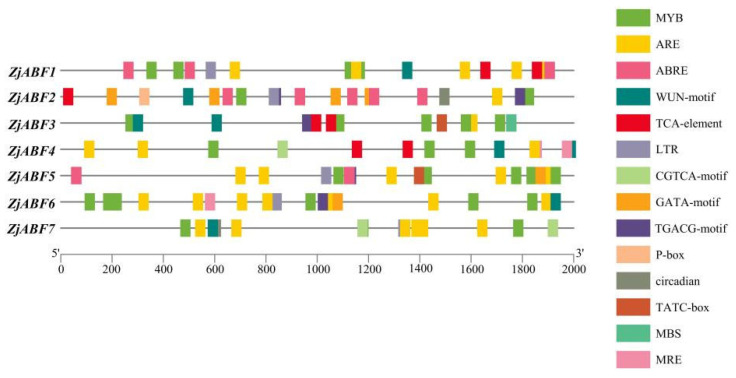
The distribution of cis-acting elements in the *ABF/AREB/ABI5* genes of *Z. jujuba* cv. Dongzao. Differently colored rectangles represent different cis-acting elements. The length and position of each *ZjABF* gene are proportionally located. The scale bar represents the length of the DNA sequence.

**Figure 8:**
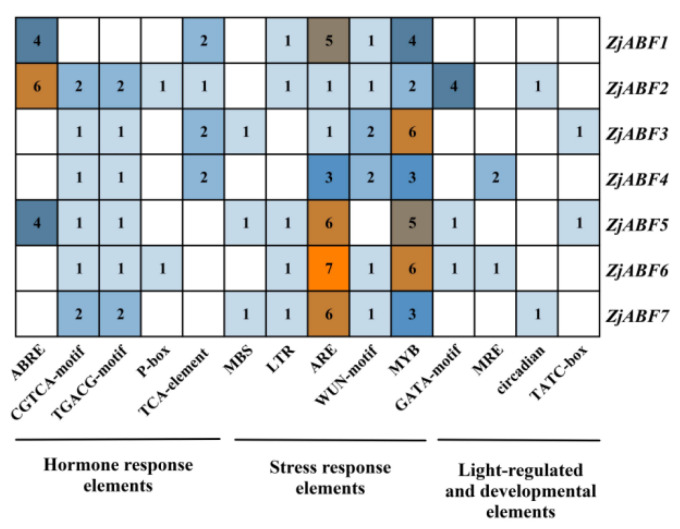
The number of cis-acting elements in the *ABF/AREB/ABI5* genes of *Z. jujuba* cv. Dongzao.

To systematically investigate the functional mechanisms of the *ABF/AREB/ABI5* genes in *Z. jujuba* cv. Dongzao, we analyzed cis-regulatory elements within the 2.0 kb upstream promoter regions using the PlantCARE database. This analysis revealed the presence of diverse elements implicated in key biological processes (Figure 7): hormone signaling—elements responsive to abscisic acid (ABA), methyl jasmonate (MeJA), salicylic acid (SA), and gibberellin (GA) (Figure 8); abiotic stress response—elements associated with drought (MBS), low temperature (LTR), anoxia (ARE), and wounding (WUN-motif); light regulation—elements involved in light signaling (GATA, MRE) and circadian control; and developmental regulation—elements conferring meristem specificity (TATC-box) and tissue specificity (GATA).

Notably, the ABA-responsive element (ABRE), characterized by a core motif similar to ACGTGGC and crucial for ABA signaling and plant responses to drought and salinity stress, was identified. *ZjABF1*, *ZjABF2*, and *ZjABF5* exhibited prominent enrichment of ABREs. Furthermore, the MYB binding site (MBS), which is involved in drought stress responses (e.g., regulating genes for stomatal closure and osmoregulation), was present in the promoters of *ZjABF3*, *ZjABF5*, and *ZjABF7*.

Collectively, the prevalence of ABRE and MBS elements within the *ZjABF* promoters strongly suggests that this gene family participates in stress adaptation regulatory networks, particularly through ABA and drought signaling pathways.

### 3.6. Z. jujuba cv. Dongzao ABF/AREB/ABI5 Gene Family Syntenic Analysis

**Figure 9:**
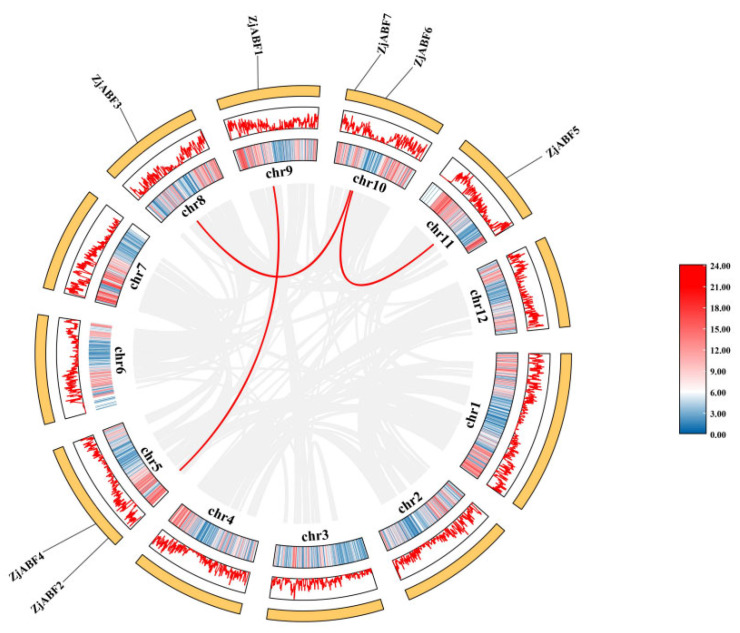
Schematic diagram of chromosome distribution and collinearity relationship of *ABF/AREB/ABI5* gene family in *Z. jujuba* cv. Dongzao. The gray and red lines represent the synteny blocks in the *Z. jujuba* cv. Dongzao genome, and the *ABF/AREB/ABI5* repeat gene pairs, respectively. The gene density heat map is in the outermost circle, shown in blue and pink.

Gene duplication is an indispensable driver in the evolution of gene families, facilitating gene amplification and the emergence of novel functional genes. Consequently, a comprehensive analysis of duplication events within the *Z. jujuba* cv. Dongzao *ABF/AREB/ABI5* gene family was conducted. Visualization using TBtools allowed for the identification of a total of three segmental duplication events among the family members (Figure 9). These duplicated genes are distributed across five chromosomes. Collectively, these findings indicate that segmental duplication events have contributed to the expansion and diversification of the *ABF/AREB/ABI5* gene family throughout the evolution of *Z. jujuba* cv. Dongzao.

### 3.7. Prediction of Z. jujuba cv. Dongzao ABF/AREB/ABI5 Protein Interactions

**Figure 10:**
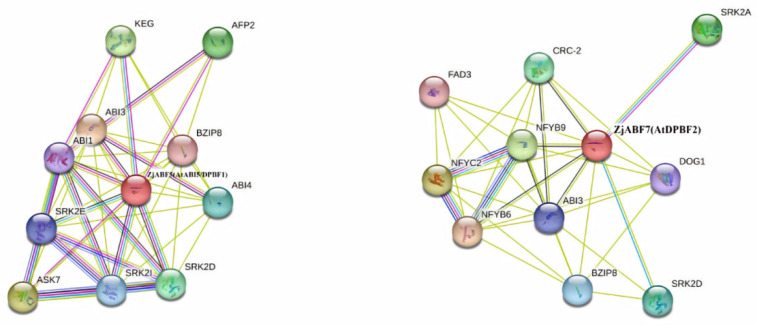
Protein network prediction of ZjABF protein function.

To predict functionally associated proteins, interaction networks for the *Z. jujuba* cv. Dongzao ABF/AREB/ABI5 proteins were constructed using the STRING database, with Arabidopsis thaliana selected as the reference species. The analysis revealed ZjABF5 and ZjABF7 as central hubs within the predicted network. Both proteins exhibited interactions with key regulators, including ABI3, BZIP8, and SRK2D (Figure 10).

Notably, *ABI3*—a downstream transcription factor in the ABA signaling pathway characterized by its B3 domain—acts as a master regulator of seed dormancy and germination. *SRK2D*—an essential component and activator within the ABA signaling cascade—broadly regulates diverse ABA responses. The predicted interactions between the ZjABF proteins (particularly ZjABF5 and ZjABF7) and these core ABA signaling components suggest direct or indirect functional associations. This strongly implicates the ABF/AREB/ABI5 family, especially ZjABF5 and ZjABF7, in mediating ABA responses and critical biological processes such as seed germination in *Z. jujuba* cv. Dongzao.

### 3.8. Tissue Expression Specificity Analysis of Z. jujuba cv. Dongzao ABF/AREB/ABI5 Family Genes

To study the functions of the *ZjABF* genes, tissue-specific expression analysis was performed on seven *ZjABF* genes, and we selected nine tissues: the flower, the young leaf, the old leaf, the young stem, the old stem, the root, the white ripe fruit, the half-red fruit, and the fully red fruit.

**Figure 11:**
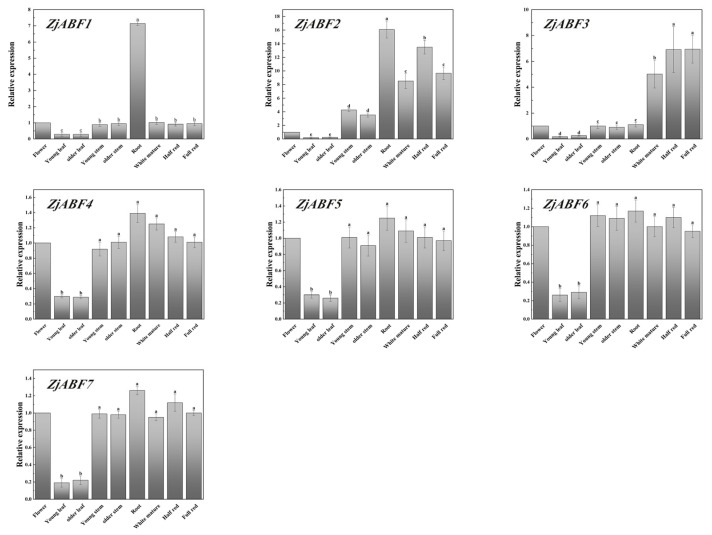
Expression analysis of seven *ZjABF* genes nine representative tissues (flowers, young leaves, old leaves, young stems, old stems, roots, white ripe stage, half-red stage, fully red stage) by qRT-PCR. The expression of the flowers was used as a control, and the 2^−∆∆Ct^ method was used to calculate the relative expression. Error bars indicate the ± standard error (SE) of three biological replicates, and a, b, c, d, e indicate the significant difference. Marked with the same letter means *p* > 0.05, and marked with different letters means *p* < 0.05.

The following key findings emerged. Most of the *ABF/AREB/ABI5* genes were highly expressed in the root; the expression of *ZjABF2* and *ZjABF3* was markedly higher in fruits at all three ripening stages (white ripe, semi-red, all-red) relative to their expression in non-fruit tissues (Figure 11). This pronounced enrichment in developing and ripening fruits suggests potential regulatory roles for these genes during fruit maturation processes.

### 3.9. Expression Analysis of the Z. jujuba cv. Dongzao ABF/AREB/ABI5 Gene Family Under Drought Stress Treatment

**Figure 12:**
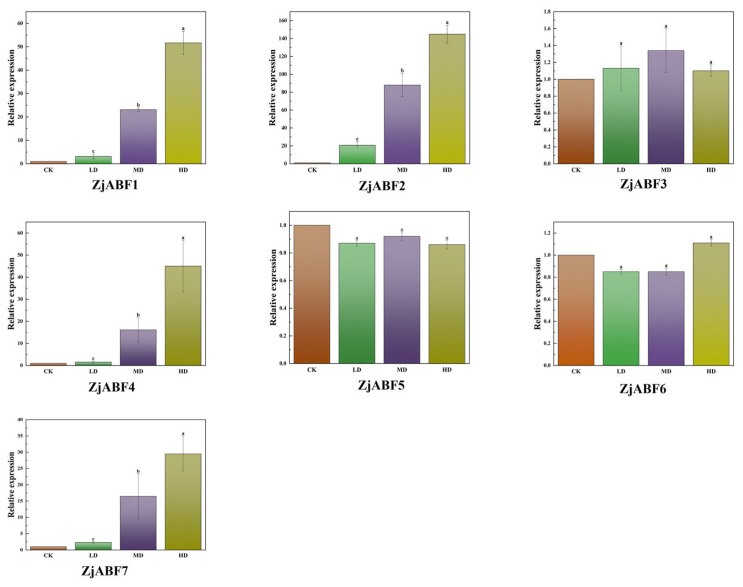
Relative expression analysis of *ABF/AREB/ABI5* genes in *Z. jujuba* cv. Dongzao under drought stress (qPCR was used to determine the expression patterns of seven *ZjABF* genes under control and mild, moderate, and severe drought stress treatments). The data are presented as the mean ± standard error (SE) of three separate measurements, and a–c indicate the significant difference. Those marked with the same letter mean *p* > 0.05, and different letters mean *p* < 0.05.

To elucidate the roles of *ABF/AREB/ABI5* genes in abiotic stress tolerance in *Z. jujuba* cv. Dongzao, the expression levels of the seven *ZjABF* genes were analyzed under drought stress treatments. The results revealed that *ZjABF1*, *ZjABF2*, *ZjABF4*, and *ZjABF7* displayed the most pronounced upregulation (Figure 12); they were 51.67 times, 144.91 times, 45.02 times, and 29.48 times the expression in the CK, respectively. Among these, *ZjABF2* emerged as the most responsive gene to drought stress.

## 4. Discussion

Abscisic acid (ABA) is a crucial plant hormone [25]. Members of the ABF/AREB/ABI5 family belong to the A subfamily of bZIP transcription factors. Characterized by their ability to specifically recognize the ABA-responsive element (ABRE) cis-acting motif in promoter regions of downstream genes, these factors participate in ABA signaling pathways and are collectively designated ABRE-binding factors (ABFs). Notably, ABI5 shares high homology with ABF/AREB members in both protein domain architecture and functional regulation, leading to its systematic inclusion within this subfamily [8]. This study identified seven *ABF/AREB/ABI5* genes in the *Z. jujuba* cv. Dongzao genome. These genes are distributed across five chromosomes and include one segmental duplication event. Comparative analysis revealed variation in family size across species: tomato harbors 10 members on five chromosomes [11], black nightshade (*Solanum nigrum*) possesses 7 members on five chromosomes [6], and poplar (*Populus trichocarpa*) contains 14 members across nine chromosomes [26]. This pattern suggests a correlation between chromosome number and *ABF/AREB/ABI5* gene family size, potentially driven by mechanisms such as whole-genome duplication (WGD) and tandem gene duplication. ABF/AREB/ABI5 family members are predominantly localized to the nucleus [27]. Consistent with findings in *Arabidopsis*, where these factors localize to the nucleus and can form heterodimers, all seven *Z. jujuba* cv. Dongzao ABF/AREB/ABI5 proteins were predicted to be nucleus-localized. This supports the hypothesis that dimerization represents an additional regulatory layer for modulating the activity of these transcription factors in *Z. jujuba* cv. Dongzao. Secondary structure analyses in adzuki bean (*Vigna angularis*) and common bean (*Phaseolus vulgaris*) indicate that AREB1 proteins are primarily composed of α-helices and random coils [28]. Similarly, our analysis of the *Z. jujuba* cv. Dongzao ABF/AREB/ABI5 family revealed α-helices and random coils as the dominant structural elements, with random coils being the most abundant. The prevalence of random coils may facilitate connections between diverse secondary structural elements within these regulatory proteins.

*Arabidopsis thaliana* ABF/AREB/ABI5 family proteins possess a conserved modular structure: the C-terminus harbors the bZIP domain responsible for DNA binding, while the N-terminus to C-terminus sequentially contains four Ser/Thr phosphorylation motifs (designated C1–C4), with C1–C3 located in the N-terminal region and C4 adjacent to the bZIP domain. The phosphorylation status of these motifs directly regulates protein activity and downstream signaling [29]. For instance, the phosphorylated active form of AREB1 can induce ectopic gene expression in vegetative tissues [30]. While a theoretical framework for phosphorylation sites in *Z. jujuba* cv. Dongzao ABF/AREB/ABI5 proteins remains to be established, our study reveals that, analogous to Arabidopsis, all *Z. jujuba* cv. Dongzao *ABF/AREB/ABI5* genes encode a distinctive BRLZ domain at the C-terminus of the bZIP region, implicated in the recognition and binding of specific DNA sequences. Beyond the highly conserved bZIP domain, ZjABF proteins also contain four conserved putative phosphorylation sites. Our motif analysis indicates that Motif 1 constitutes the core bZIP domain. Furthermore, a complete phosphorylation regulatory network is formed by Motif 5 and Motif 2 (corresponding to the C1 site), Motif 3 (C2 site), Motif 4 (C3 site), and Motif 6 (C4 site). Analysis of gene structure demonstrates that *Z. jujuba* cv. Dongzao *ABF/AREB/ABI5* genes contain between three and five exons and two to six introns. This organization is highly similar to the genetic structures observed in potato (*Solanum tuberosum*) and poplar [26], confirming the conserved gene composition of ABF/AREB/ABI5 family members in *Z. jujuba* cv. Dongzao.

*A. thaliana* ABF/AREB/ABI5 subfamily members exhibit tissue-specific expression during vegetative growth, with transcript activity significantly enriched in vegetative organs. Notably, AREB1/ABF2, AREB2/ABF4, and ABF3 function cooperatively to regulate ABRE-dependent ABA signaling, playing critical roles in tolerance to drought stress [31]. Phylogenetically, *ZjABF1* and *ZjABF2* cluster within Group C, alongside the Arabidopsis orthologs AREB1/ABF2, AREB2/ABF4, and ABF3. Mirroring the functional implications of this grouping, *ZjABF1* and *ZjABF2* display pronounced expression in roots, while *ZjABF2* and *ZjABF3* are highly expressed during fruit maturation. Furthermore, *ZjABF1* and *ZjABF2* exhibit the most significant induction under drought stress. These expression patterns, coupled with their phylogenetic placement, strongly suggest that Group C *ZjABF* genes function similarly to their Arabidopsis counterparts, likely playing pivotal roles in ABA-mediated stress response pathways in *Z. jujuba* cv. Dongzao. Additionally, these ABF/AREB/ABI5 transcription factors share similarities in subcellular localization, gene structure, and tissue expression profiles. Intriguingly, *ZjABF4* shares cell localization and gene structure characteristics with *ZjABF1* and *ZjABF2* and uniquely possesses Motif 7 alongside them. This combination of shared features suggests that *ZjABF4* may represent a functional homolog of *ZjABF1* and *ZjABF2*.

Cis-regulatory elements within gene promoters serve as core hubs in plant signaling networks, integrating environmental cues and modulating downstream gene expression by specifically binding transcription factors. As key regulatory nodes, ABF/AREB/ABI5 family members are widely implicated in plant responses to abiotic stresses (e.g., drought, salinity, cold) and developmental regulation [32]. *Arabidopsis ABF/AREB/ABI5* gene expression is coordinately induced by light signals, ABA, and multiple abiotic stresses (drought, high salinity, and cold), forming a multi-layered stress response network [33]. Beyond these signals, *PmABF*s in *Prunus mume* (Rosaceae) can also be activated by exogenous hormones (e.g., gibberellins, ethylene) and hypoxia stress, implying roles in broader physiological adaptation [34]. Consistent with this regulatory complexity, our analysis of *ZjABF* promoters reveals an abundance of cis-elements associated with hormone signaling, abiotic stress responses, and light regulation, underscoring their critical functions in the growth, development, and stress adaptation of *Z. jujuba* cv. Dongzao.

In this study, most of the *ABF/AREB/ABI5* genes were highly expressed in the root. Under drought stress, both *ZjABF1, ZjABF2, ZjABF4* and *ZjABF7* were upregulated, and *ZjABF2* contained the most ABRE cis-reactive elements, suggesting that the water-stress-responsive members of the AREB/ABF/ABI5 subfamily regulate the ABRE-dependent ABA signaling involved in drought stress tolerance. At the same time, they were highly expressed in the root. Based on this, we speculated that ABA is primarily synthesized in the root and then transported to the target tissues by the xylem and phloem, identifying the transport pathway between the root and shoot of *Z. jujuba* cv. Dongzao. Our findings agree with those regarding *Arabidopsis* [31].

## 5. Conclusions

This study conducted genome-wide identification of the ABF/AREB/ABI5 transcription factor family in *Z. jujuba* cv. Dongzao. Employing bioinformatic analyses, we characterized the structural features, physicochemical properties, and predicted gene functions of the identified family members. Expression profiling under graded drought stress revealed candidate genes which are responsive to water deficits. Notably, *ZjABF2* emerged as a pivotal regulator, being implicated in both fruit maturation processes and adaptation to drought stress. These findings establish a foundation for elucidating the molecular mechanisms underlying drought tolerance mediated by *ABF/AREB/ABI5* genes in jujube and highlight *ZjABF2* as a prime candidate for further functional investigation.

## Figures and Tables

**Table 1:** Physical and chemical property analysis and subcellular localization prediction of ABF/AREB/ABI5 family members in *Z. jujuba* cv. Dongzao.

Gene Name	Gene ID (NCBI)	Number of Amino Acids	Molecular Weight	Theoretical pI	Instability Index	Aliphatic Index	Grand Average of Hydropathicity	Prediction of Subcellular Location
*ZjABF1*	*LOC107427086*	436	46235.87	9.85	52.33	66.93	−0.614	Nuclear
*ZjABF2*	*LOC107420002*	470	50760.93	7.75	35.62	68.09	−0.592	Nuclear
*ZjABF3*	*LOC107412942*	321	35584.09	8.50	59.61	66.88	−0.807	Nuclear
*ZjABF4*	*LOC107422670*	325	36397.92	5.39	51.58	80.12	−0.534	Nuclear
*ZjABF5*	*LOC107432186*	438	48065.62	8.47	61.51	63.88	−0.787	Nuclear
*ZjABF6*	*LOC107432156*	288	32464.47	9.32	48.95	76.28	−0.739	Nuclear
*ZjABF7*	*LOC107409642*	474	52642.00	8.82	55.84	64.58	−0.798	Nuclear

**Table 2:** The secondary structure of the *ABF/AREB/ABI5* gene family protein sequence in *Z. jujuba* cv. Dongzao. Blue represents the alpha spiral; purple represents the extended chain; orange represents irregular curls.

Protein	Alpha Helix (%)	Extended Strand (%)	Random Coil (%)	Distribution of Secondary Structure Elements
ZjABF1	28.21%	0.69%	71.10%	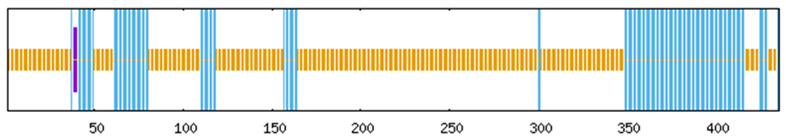
ZjABF2	34.47%	3.19%	62.34%	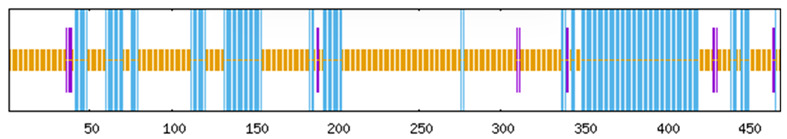
ZjABF3	35.20%	0.00%	64.80%	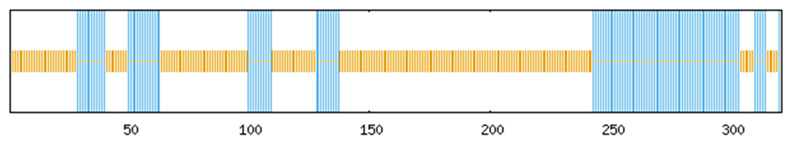
ZjABF4	36.00%	1.23%	62.77%	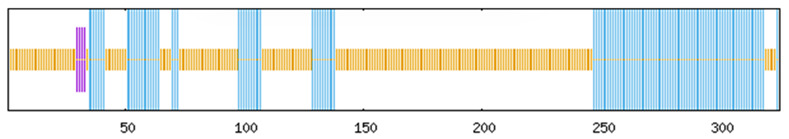
ZjABF5	30.37%	1.60%	68.04%	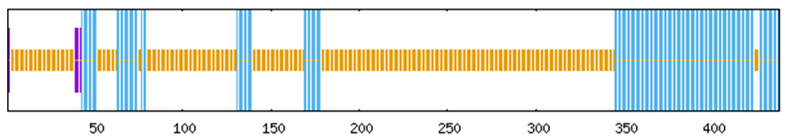
ZjABF6	46.88%	2.78%	50.35%	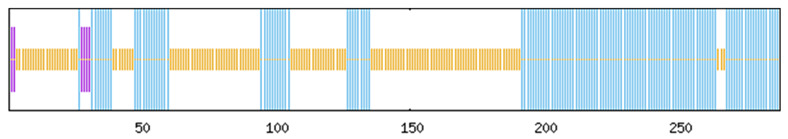
ZjABF7	31.65%	4.43%	63.92%	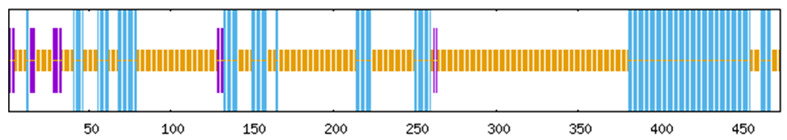

## Data Availability

The original contributions presented in this study are included in the article. Further inquiries can be directed to the corresponding author.
